# Supplementary material for: Osteonecrosis of the Femoral Head: A Multidisciplinary Approach in Diagnostic Accuracy
Source: Diagnostics (Basel). 2022 Jul 16;12(7):1731. doi: 10.3390/diagnostics12071731 (PMC9324583; doi:10.3390/diagnostics12071731)
Supplement: Supplementary file 1 [file diagnostics-12-01731-s001.zip › diagnostics-1776802-supplementary.pdf]

Supplementary Table S1.

Supplementary Table S1. Anatomopathological exam from selected bone samples. Femoral head specimens underwent histological analysis pondering: the average percentage of empty osteocyte lacunae (at least 300 lacunae were quantified in four non-overlapping regions of interest) and the presence/absence of heterotopic ossification (dystrophic calcification present in H&E staining). Fatty infiltration and medullary fibrosis were graded in qualitative manner ("1-absence", "2-presence", "3-moderate", and "4-intense"), being assessed by H&E and Masson trichrome staining respectively. A statistically significant difference in the number of empty osteocytic lacunae ($t=5.13$; $p<0.05$) was found between the ONFH (mean ± SE = 68,16 ± 10,57) and the OA group (mean ± SE = 40,91 ± 15,03). There was also a statistically significant difference ($U=35$; $p<0.05$) between medullary fibrosis presence in the ONFH (mean ± SE = 2,75 ± 1,21) and the OA (mean ± SE = 1,66 ± 0,98) group. Fatty infiltration and heterotopic ossification showed no statistically significant differences between both groups.

Specimen	Empty lacunae (%)	Fatty Infiltration (1-4)	Heterotopic Ossification (presence)	Fibrosis (1-4)
1 (CASE)	64	4	NO	4
2 (CASE)	75	4	NO	2
3 (CASE)	57	4	NO	1
4 (CASE)	80	4	NO	4
5 (CASE)	73	4	NO	4
6 (CASE)	71	2	NO	1
7 (CASE)	74	4	NO	2
8 (CASE)	70	2	YES	3
9 (CASE)	42	4	NO	2
10 (CASE)	63	2	NO	2
11 (CASE)	70	4	YES	4
12 (CASE)	79	4	YES	4
13 (CONTROL)	61	2	NO	2
14 (CONTROL)	47	3	NO	1
15 (CONTROL)	40	2	NO	1
16 (CONTROL)	44	4	YES	4
17 (CONTROL)	47	2	NO	1
18 (CONTROL)	54	3	NO	1
19 (CONTROL)	25	4	NO	1
20 (CONTROL)	41	3	YES	3
21 (CONTROL)	47	3	NO	1
22 (CONTROL)	52	2	NO	1
23 (CONTROL)	27	4	YES	2
24 (CONTROL)	6	2	NO	2
